# Enzymatic Fat Dissolution Improves Detection of Small Lymph Nodes in Colon Cancer Surgery

**DOI:** 10.7759/cureus.53792

**Published:** 2024-02-07

**Authors:** Ryuji Kajitani, Taro Munechika, Yoshiko Matsumoto, Hideki Nagano, Naoya Aisu, Gumpei Yoshimatsu, Yoichiro Yoshida, Suguru Hasegawa

**Affiliations:** 1 Gastroenterological Surgery, Fukuoka University Hospital, Fukuoka, JPN

**Keywords:** lymph node size, surgery, enzymatic fat dissolution, lymph node evaluation, colon cancer

## Abstract

Background

Accurate lymph node evaluation is essential for staging colon cancer and guiding postoperative treatment decisions. In this study, we compared the efficacy of a simple enzymatic fat dissolution method with the conventional method for lymph node sampling from specimens after colon cancer surgery.

Methods

We enrolled 58 patients who underwent elective laparoscopic surgery for colon adenocarcinoma between May 2018 and May 2021 at Fukuoka University Hospital in Fukuoka, Japan. The specimens from these patients were treated using fat dissolution and were compared with specimens from 58 patients for which conventional manual palpation was used.

Results

A significantly greater number of lymph nodes were detected by the fat dissolution method compared with the conventional method (average per patient, 27.5 vs. 22.6, P = 0.02). In particular, the between-group difference was significant for lymph nodes measuring <5 mm (average per patient, 26.1 vs. 20.9; P = 0.01). Multivariate analysis showed that, compared with the conventional method, the fat dissolution method was significantly associated with the identification of lymph node metastasis. The positive rate of lymph nodes ≥10 mm in diameter was markedly higher along the inferior mesenteric artery than the ileocolic artery (100% vs. 52.6%).

Conclusions

The use of the fat dissolution method led to an increase in the number of small lymph nodes detected. Rates of metastasis according to lymph node size may depend on the lymph node station.

## Introduction

Accurate histological lymph node staging is crucial for estimating prognosis and making treatment decisions in patients with colorectal cancer. In accordance with the TNM classification for colorectal cancer in the 8th edition of the American Joint Committee on Cancer (AJCC) staging manual, the stage of colorectal cancer is determined by the number of metastatic lymph nodes [1,2]. When the positive lymph nodes are confirmed pathologically after surgical resection, adjuvant chemotherapy is recommended as stage III colorectal cancer, based on the clinical phase 3 trials [1,2]. However, harvesting lymph nodes through manual palpation of the specimen before formalin fixation in Japan and after tissue fixation in Europe has some limitations, especially in terms of detecting small lymph nodes [3]. Hernanz et al. found that, on average, 10 lymph nodes per specimen were missed by fat clearance after traditional dissection [4]. Missed lymph nodes can lead to understaging of the cancer, noncompliance with adjuvant chemotherapy, and worsening of prognosis. Therefore, various methods have been developed to reduce the risk of missed lymph nodes [5], but many of these methods require the use of harmful organic solvents and are complicated and time-consuming [6].

In this study, we compared the efficacy of a simple enzymatic fat dissolution (FD) method that does not require the use of organic solvents for lymph node sampling in specimens after colon cancer surgery with that of the conventional method. Considering that the size criterion for a metastasis-positive lymph node in the colon is widely thought to be 10 mm and does not take into account differences by the tumor or vessel site [7], we also investigated whether the frequency of metastasis according to lymph node size varies depending on the station.

This article was previously posted to the ResarchGate preprint server on October 2023.

## Materials and methods

Patients

This is a prospective cohort study. We enrolled 58 patients who underwent elective laparoscopic surgery for adenocarcinoma of the colon (cecum to sigmoid colon) between May 2018 and May 2021 at Fukuoka University Hospital in Fukuoka, Japan, in whom resected specimens were treated with the FD method for lymph node retrieval. These patients were assigned to the FD group. A further 58 patients who underwent the same surgery between January 2016 and March 2017, in whom the lymph nodes were detected manually by a surgeon without formalin fixation of the tissue after surgery, were enrolled in the conventional group. The inclusion criteria were age ≥20 years, elective surgery, and provision of written informed consent. Patients with tumors in which the evaluation of lymph nodes was considered difficult, those with a history of metachronous cancers within the previous five years, and those patients with multiple cancers were excluded.

The study was approved by the Institutional Review Board of Fukuoka University Hospital, Fukuoka, Japan, registered in the UMIN Clinical Trials Registry (17-8-01), and conducted in accordance with the tenets of the Declaration of Helsinki. Written informed consent for enrollment was obtained from all study participants. 

Study protocol

**Figure 1:**
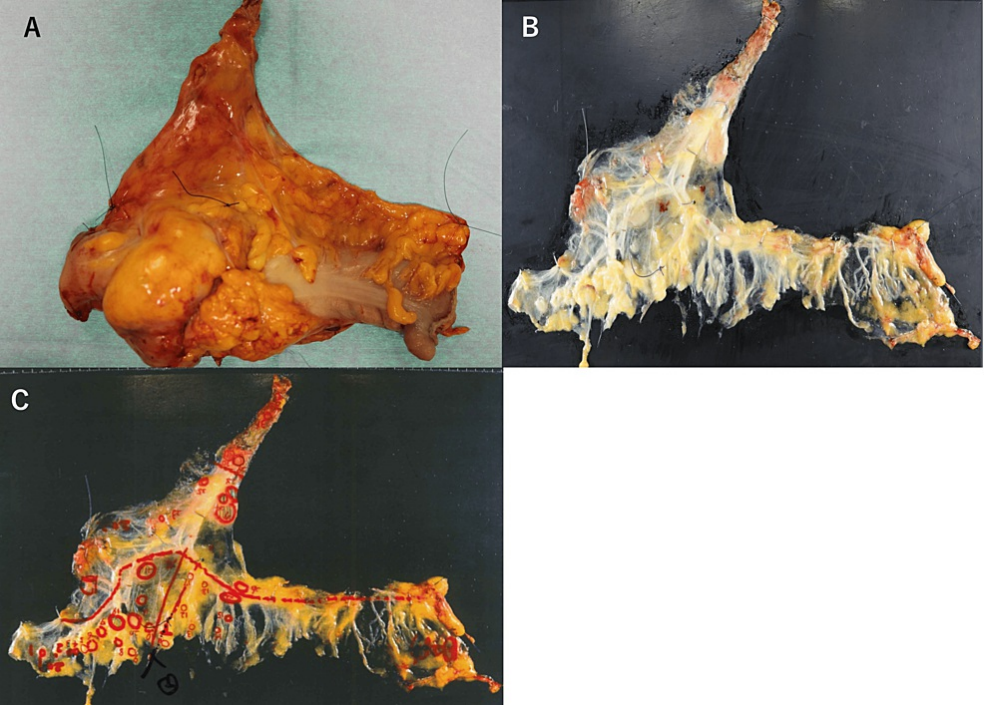
Specimen after colon cancer surgery A: Specimen after colon cancer surgery. B: After fat dissolution. C: The location of lymph nodes and their positional relationship with blood vessels were recorded.

After excision of the specimen, only the mesentery was dissected from the intestine without fixation. The mesentery was then injected with FD solution (Imofully®; Sysmex Corporation, Kobe, Japan) containing lipase and trypsin using a 22-G needle. The solution-infused mesentery was attached to a board, wrapped in a bag, and immersed in water at 40°C for approximately one hour. Next, the melted fat in the mesentery was wiped off with gauze, and lymph nodes were identified. At that time, the location and size of the lymph nodes and their positional relationship with blood vessels were recorded in detail. The short diameter was used as the size of the lymph node (Figure 1).

Study endpoints

The primary endpoint of the study was the difference in the number of harvested and metastatic lymph nodes between the conventional and FD groups, particularly according to the size of the lymph node. The secondary endpoint was the size of the lymph nodes (metastatic or non-metastatic) according to the lymph node station. Lymph node metastasis was graded according to the 8th edition of the Union for International Cancer Control TNM classification.

Statistical analysis

The data are shown as the median (interquartile range) or number (percentage) and were compared between groups, using the Mann-Whitney U test or chi-squared test. Multivariate logistic regression analysis with adjustment was performed to determine the effect of detecting lymph node metastasis. All statistical analyses were performed using JMP Pro version 17.0 for Macintosh (SAS Institute Inc., Cary, NC). A p-value <0.05 was considered statistically significant.

## Results

Background characteristics

The patient characteristics are shown in Table 1. Fifty-eight patients each were enrolled in the FD and conventional groups. There was no significant between-group difference in age, sex, or body mass index. D3 dissection was performed in all patients in both groups, and there was no significant difference in the length of the resected bowel. Pathological factors are shown in Table 2. There were no significant differences in histological type, lymphovascular invasion, T stage, or N stage between the two groups.

**Table 1:** Patient characteristics *The values represent the median and interquartile range. **C: cecum, A: ascending colon, T: transverse colon, D: descending colon, S: sigmoid colon

		Fat dissolution group	Conventional group	
		n=58	n=58	P-value
Age (years)		71.5 (66.3-77)*	72 (65.5-77.5)*	0.56
Sex (M/F)		23/35	22/36	0.84
Tumor location**				
	C/A/T	3/27/14	7/29/15	0.32
	D/S	10/4	6/1	
Body mass index		21.3 (19.6-23.1)*	22.3 (20.0-24.1)*	0.11
D3 lymph node dissection		58	58	-
Length of specimen (mm)		190 (160-220)*	210 (180-237)*	0.23
Operative procedure				
	Ileocecal/right colectomy	21/13	19/19	0.21
	Left colectomy/transverse colectomy	9/15	3/17	

**Table 2:** Pathological outcomes *The values represent the median and interquartile range. **Pathological T stage by the National Comprehensive Cancer Network (NCCN) Guidelines. ***Pathological N stage by the NCCN Guidelines.

	Fat dissolution group	Conventional group	P-value
	n=58	n=58	
Tumor diameter (mm)	45 (31-60)*	50 (30-60)*	0.84
Histology			
Well, moderate	51	55	0.18
Poorly, mucinous	7	3	
pT factor**			
T2	4	5	0.72
T3, T4a	54	53	
Lymphatic invasion	40	31	0.08
Vascular invasion	48	47	0.8
pN factor***			
N0	30	37	0.4
N1	20	16	
N2	7	5	
N3	1	0	

Effect of FD on the number of lymph nodes identified

The total number of lymph nodes identified was 1,597 in the FD group (n = 58) and 1,316 in the conventional group (n = 58) (Table 3). The average number of lymph nodes identified per patient was significantly greater in the FD group than in the conventional group (27.5 vs. 22.6; P = 0.02). The lymph nodes were divided into three groups according to whether their diameter was <5 mm, ≥5 mm to <10 mm, or ≥10 mm. The number of lymph nodes measuring <5 mm was 1,511/1,597 (94.6%) in the FD group and 1,214/1,316 (92.2%) in the conventional group. A significantly greater average number of lymph nodes measuring <5 mm per patient was identified in the FD group than in the conventional group (average 26.1 vs. 20.9; P = 0.01).

**Table 3:** Number of lymph nodes found by short diameter (per patient) Values are expressed as the mean (± standard deviation).

		Fat dissolution group (n = 58)	Conventional group (n = 58)	P-value
Total				
	<5 mm	26.1 ± 1.6	20.9 ± 1.1	0.01
	5-10 mm	1.1 ± 0.2	1.5 ± 0.2	0.1
	>10 mm	0.3 ± 0.1	0.2 ± 0.1	0.16
	Total	27.5 ± 1.7	22.6 ± 1.2	0.02
Metastatic				
	<5 mm	0.86 ± 0.2	0.51 ± 0.2	0.23
	5-10 mm	0.31 ± 0.1	0.33 ± 0.1	0.88
	>10 mm	0.26 ± 0.1	0.09 ± 0.05	0.1
	Total	1.43 ± 0.3	0.93 ± 0.2	0.17

Effect of FD on the rate of detection of metastatic lymph nodes

The results for metastatic lymph nodes are shown in Table 3. There was no significant difference in the average total number of positive lymph nodes per patient between the FD group and the conventional group (1.43 vs. 0.93; P = 0.17) or in the average number of lymph nodes measuring <5 mm (0.86 vs. 0.51; P = 0.23). Factors associated with metastatic lymph nodes are shown in Table 4. Univariate analysis identified lymph node size, lymph node group, T stage, and lymphovascular invasion to be significantly associated with metastasis to lymph nodes. Logistic regression analysis with adjustment for these factors showed an independent association between FD and lymph node metastasis.

**Table 4:** Factors associated with lymph node metastasis *N = total number of lymph nodes. † metastasis = total number of metastasis lymph nodes. ‡ odds ratio and 95% confidence interval. § LN = lymph node

			Univariate						Multivariate			
	N*		Metastasis^†^		Odds ratio^‡^		P-value		Odds ratio^‡^		P-value	
Sex							0.12					
Male	1091		60 (5.5%)		Ref.							
Female	1822		77 (4.2%)		0.76 (0.54-1.07)							
Age, years							0.31					
<75	1737		76 (4.4%)		Ref.							
≥75	1176		61 (5.2%)		1.19 (0.84-1.68)							
Body mass index							0.41					
<25	2571		116(4.5%)		Ref.							
≥25	342		19 (5.5%)		1.24 (0.75-2.04)							
Size of LN^§^							<0.001				<0.001	
<5 mm	2725		80 (2.9%)		Ref.				Ref.			
5-10 mm	156		37 (23.7%)		10.3 (6.68-15.8)		<0.001		14.5 (8.9-23.7)			
≥10 mm	32		20 (62.5%)		55.1 (26.0-116)		<0.001		107 (43-264)			
LN^§^ group							<0.001				<0.001	
Paracolic	1617		97 (6%)		Ref.				Ref.			
Intermediate	801		29 (3.6%)		0.59 (0.39-0.90)		0.014		0.43 (0.26-0.70)			
Apical	495		11 (2.2%)		0.36 (0.19-0.70)		0.001		0.38 (0.19-0.76)			
pT							<0.001				<0.001	
pT1/2	1383		29 (2.1%)		Ref.				Ref.			
pT3/4a/4b	1530		108 (7.1%)		3.55 (2.34-5.38)				7.3 (4.2-12.7)			
Lymphatic invasion							<0.001				<0.001	
ly-	1202		20 (1.6%)		Ref.				Ref.			
ly+	1711		117 (6.8%)		4.33 (2.68-7.01)				7.1 (3.6-14.0)			
Venous invasion							0.0017				<0.001	
v-	608		15 (2.5%)		Ref.				Ref.			
v+	2305		122 (5.3%)		2.21 (1.28-3.8)				4.6 (2.0-10.4)			
Method							0.16				<0.001	
Conventional	1316		54 (4.1%)		Ref.				Ref.			
Fat dissolution	1597		83 (5.2%)		1.28 (0.90-1.82)				2.6 (1.7-4.1)			

Size-dependent lymph node metastasis rate according to location

The size-dependent rates of lymph node metastasis along the ileocolic artery (ICA), middle colic artery (MCA), and inferior mesenteric artery (IMA) are shown in Table 5. Examination of both groups together (FD and conventional) showed that the positive rates for large lymph nodes measuring ≥10 mm varied by lymph node station, with 52.6% (10/19) along the ICA, 40.0% (2/5) along the MCA, and 100.0% (5/5) along the IMA (P = 0.04). In addition, the number of large non-metastatic lymph nodes (≥5 mm) per case also significantly differed by vessel, with 1.30 along the ICA, 0.36 along the MCA, and 0.5 along the IMA (P < 0.001).

**Table 5:** Relationship between the lymph node size and metastasis by lymph node region ICA: ileocolic artery, MCA: middle colic artery, IMA: inferior mesenteric artery, LN: lymph node

	Diameter	ICA (73 cases)	MCA (46 cases)	IMA (27 cases)	P-value
Metastatic rate					
	<5 mm	49/1488 (3.3%)	12/533 (2.3%)	13/470 (2.8%)	0.44
	≥5 mm to <10 mm	25/111 (22.5%)	6/20 (30%)	4/18 (22.2%)	0.77
	≥10 mm	10/19 (52.6%)	2/5 (40%)	5/5 (100%)	0.04
Number of non-metastatic LNs per case					
	<5 mm	19.7	11.3	16.9	<0.001
	≥5 mm to <10 mm	1.18	0.3	0.5	<0.001
	≥10 mm	0.12	0.06	0	0.122
	≥5 mm	1.3	0.36	0.5	<0.001

## Discussion

In this study, we found that the FD method increased the number of harvested lymph nodes, particularly those that were small, and prevented positive lymph nodes from being missed, as well as the understaging of tumors. Moreover, this method is simple to use and less biohazardous compared with conventional FD methods that use organic solvents [6].

In the US and UK, it is recommended that at least 12 lymph nodes be found in postoperative colorectal cancer specimens. Finding fewer than 12 lymph nodes has been associated with poorer overall survival, likely as a result of overlooking lymph node metastasis and subsequent failure to administer adjuvant chemotherapy [8-10]. Various methods have been used to improve the detection of lymph nodes. Several techniques can be used for lymph node dissection, but they often involve the use of organic solvents or are complex and require more than one day to perform [4,5]. A recent meta-analysis of randomized controlled trials reported that the use of methylene blue and FD was highly effective for harvesting lymph nodes [11]. However, evidence for the efficacy of the FD method for lymph node retrieval, as in the present study, is mixed [12,13].

In our study, the number of lymph nodes harvested was significantly greater in the FD group than in the conventional group, indicating that FD is effective. Previous reports have shown that the average number of lymph nodes harvested in stage II and III colon cancer is 19.4 [14]. The average number of lymph nodes in our study was 25.1, which is comparable to previous reports. Although there have been similar reports on the number of lymph nodes dissected, there have been almost no comparisons of the size of lymph nodes between the conventional and FD methods. In our study, we measured the short diameter of the excised lymph nodes in the FD group and compared it with that obtained using the conventional method. The number of lymph nodes with a diameter of ≤5 mm was found to be greater in the FD group than in the conventional group, suggesting that FD is effective in preventing small lymph nodes from being overlooked.

We found that 60.2% of positive lymph nodes in the FD group and 55.6% of those in the conventional group had a diameter of ≤5 mm. Previous studies have shown that even small lymph nodes have the potential to be positive, with some reporting up to 69% of positive lymph nodes being ≤5 mm or less [15,16]. This suggests that using the FD method can help prevent understaging of colon cancer, consistent with the findings of previous studies [12]. Multivariate analysis revealed that the lymph node size, lymph node group, T stage, lymphovascular invasion, and use of the FD method were significantly associated with lymph node metastasis. While univariate analysis did not show a significant difference, multivariate analysis demonstrated that the use of FD was significantly associated with the discovery of lymph node metastasis.

We compared the size of lymph nodes along the ICA, MCA, and IMA and found that large non-metastatic lymph nodes (≥5 mm) are found more frequently along the ICA than along the MCA or IMA. Furthermore, the rate of positivity of lymph nodes ≥10 mm in size was lower in the ICA area than in the IMA area. These findings suggest that lymph nodes along the ICA may not metastasize even if they are large in size and that the criteria for metastasis-positive lymph node size should be considered for each blood vessel in computed tomography scans. This is important for determining the indications for preoperative chemotherapy [17].

This study has several limitations, including the small number of patients, an unclear long-term prognosis, and the inability to perform a blind test for this intervention. Nevertheless, we believe that the results of this study might contribute to improving the accuracy of diagnosing lymph node metastasis.

## Conclusions

This study demonstrated that the FD method was effective in increasing the number of picking up lymph nodes from the dissected tissue, especially small ones, and that it could prevent the understaging of colon cancer. Precise pathological staging impacts the decision of applying adjuvant chemotherapy appropriately, and it may improve overall survival. Our findings also identified that the size of non-metastatic lymph nodes varies depending on their location along different blood vessels. We suggest that the location of the lymph nodes should be considered to determine the preoperative clinical lymph node staging in colon cancer.
